# Regulation of endogenous hormone and miRNA in leaves of alfalfa (*Medicago sativa* L.) seedlings under drought stress by endogenous nitric oxide

**DOI:** 10.1186/s12864-024-10024-8

**Published:** 2024-03-01

**Authors:** Qian Ruan, Xiaoming Bai, Yizhen Wang, Xiaofang Zhang, Baoqiang Wang, Ying Zhao, Xiaolin Zhu, Xiaohong Wei

**Affiliations:** 1 College of Life Science and Technology, Gansu Agricultural University, Lanzhou, Gansu 730070 China; 2 Pratacultural College, Gansu Agricultural University, Lanzhou, Gansu 730070 China; 3 College of agronomy, Gansu Agricultural University, Lanzhou, Gansu 730070 China; 4 Gansu Key Laboratory of Crop Genetic Improvement and Germplasm Innovation, Lanzhou, Gansu 730070 China; 5 Gansu Key Laboratory of Arid Habitat Crop Science, Lanzhou, Gansu 730070 China

**Keywords:** Alfalfa, Drought stress, Endogenous hormone, High-throughput sequencing, NO, miRNAs

## Abstract

**Background:**

Alfalfa *(Medicago sativa*. L) is one of the best leguminous herbage in China and even in the world, with high nutritional and ecological value. However, one of the drawbacks of alfalfa is its sensitivity to dry conditions, which is a global agricultural problem. The objective of this study was to investigate the regulatory effects of endogenous nitric oxide (NO) on endogenous hormones and related miRNAs in alfalfa seedling leaves under drought stress. The effects of endogenous NO on endogenous hormones such as ABA, GA3, SA, and IAA in alfalfa leaves under drought stress were studied. In addition, high-throughput sequencing technology was used to identify drought-related miRNAs and endogenous NO-responsive miRNAs in alfalfa seedling leaves under drought stress.

**Result:**

By measuring the contents of four endogenous hormones in alfalfa leaves, it was found that endogenous NO could regulate plant growth and stress resistance by inducing the metabolism levels of IAA, ABA, GA3, and SA in alfalfa, especially ABA and SA in alfalfa. In addition, small RNA sequencing technology and bioinformatics methods were used to analyze endogenous NO-responsive miRNAs under drought stress. It was found that most miRNAs were enriched in biological pathways and molecular functions related to hormones (ABA, ETH, and JA), phenylpropane metabolism, and plant stress tolerance.

**Conclusion:**

In this study, the analysis of endogenous hormone signals and miRNAs in alfalfa leaves under PEG and PEG + cPTIO conditions provided an important basis for endogenous NO to improve the drought resistance of alfalfa at the physiological and molecular levels. It has important scientific value and practical significance for endogenous NO to improve plant drought resistance.

**Supplementary Information:**

The online version contains supplementary material available at 10.1186/s12864-024-10024-8.

## Background

One of the most important problems threatening agricultural cultivation is drought in many parts of the world [1]. Drought stress is a common adverse environmental condition. It has adverse effects on plant metabolic processes including stomatal movement, nutrient absorption, and photo compound production, and ultimately leads to crop loss [2–4]. Plants use drought avoidance and/or drought tolerance mechanisms to cope with drought. Drought is usually avoided by morphological changes in plants, such as reduced stomatal conductance, reduced leaf area, and expanded root development [5]. On the other hand, plant hormones such as cytokinin, gibberellin, auxin, and abscisic acid can be accumulated to regulate resistance to drought conditions or they can regulate drought stress-related genes [6–8]. With the discovery of small RNAs, more attention has been focused on the importance of post-transcriptional gene regulation by miRNAs in response to drought stress [9, 10]. Therefore, focusing on these plant hormones and drought-related miRNAs may be a good way to conserve and improve productivity in response to drought stress. More recently, miRNAs have become important regulators of drought tolerance and avoidance by controlling the expression of drought response genes [11, 12].

More and more data suggest that microRNAs (miRNAs) help regulate the resilience of plants [13, 14]. Specifically, miRNAs were typically 20-24nt long and bind to partially complementary sequences in the target messenger RNA (mRNA) and inhibit gene expression at the transcriptional and post-transcriptional levels through transcriptional fragment cleavage and translational inhibition [15, 16]. Drought stress was a common adverse environmental condition. It has harmful effects on the metabolic processes of plants, such as stomatal movement, nutrient absorption and the production of photosynthetic assimilates [2, 3]. However, plants have their defense systems to deal with adverse climatic conditions. One such defense mechanism is through reprogramming of gene expression through miRNAs [17]. Plant miRNAs regulate physiological processes such as auxin signal transduction, leaf morphogenesis, flowering time, and embryo development [18, 19]. A growing number of studies support the important role of miRNAs in coordinating important agronomic traits in crops. Recent studies have also reported the key role of miRNAs in drought stress adaptation in plants [20]. This makes miRNAs promising candidates for the molecular breeding of crops with enhanced drought resistance. Several drought-related miRNAs have been identified in Arabidopsis, cowpea, rice, tobacco, and soybean using global expression profiling [21–25]. In Arabidopsis, overexpression of miR164b enhances tolerance to drought stress [26]. miR408 targets copper-related genes, and overexpression of miR408 can improve the drought resistance of chickpeas [27]. Reports on genome-wide studies of alfalfa and the screening and identification of drought-resistant and salt-resistant miRNAs in model plant *Medicago truncatula* provide a new opportunity to study the regulatory mechanism of drought-resistant miRNAs in alfalfa at the molecular level [28]. The accumulation of the above studies provides the theoretical basis and research ideas for us to explore the expression mechanism of miRNAs induced by endogenous NO in alfalfa drought resistance.

As a signaling substance, NO has been widely concerned [29]. NO not only plays an important role in plant stress response [30], but also participates in the regulation of plant seed germination, root and leaf growth and development, photomorphosis, cell proliferation, differentiation and apoptosis, plant lignification, and other processes [31]. cPTIO, a NO scavenger, can significantly reduce the NO content in plant leaves and has been widely used to reveal the function of NO in plant stress resistance [32]. However, the potential mechanism by which endogenous NO enhances drought stress tolerance of alfalfa remains unclear.

Alfalfa is one of the forage grasses with the largest planting area in the world. It is known as the “king of forage grasses” because of its advantages of high protein content, rich nutrition, good palatability, and high yield [33]. Because alfalfa has a very developed root system, it can not only effectively prevent soil erosion, but also has a strong nitrogen fixation effect [34]. Research has shown that the nitrogen fixation ability of alfalfa increases the nitrogen fertilizer and organic matter in the soil at the depth of 0–40 cm (the distribution depth of alfalfa root system) in saline-alkali land by about 50%. In addition, alfalfa has a certain tolerance to heavy metal soil, and the growth of alfalfa in low-dose heavy metal soil is not inhibited, but also promotes to a certain extent, and has the ability to biological remediation of soil. Alfalfa has been widely cultivated around the world and has been identified as the main feed crop [35]. The quality of alfalfa plays a very important role in ecological environment restoration and the development of animal husbandry in China. However, in the arid areas of northwest China where alfalfa was widely cultivated, drought seriously affects the yield and quality of alfalfa and greatly restricts the development of the alfalfa industry [36]. Therefore, how to reduce the impact of drought on alfalfa production is one of the important scientific problems to be solved urgently.

## Results

### Changes of NO content in leaves of alfalfa seedlings under different treatments

**Fig. 1:**
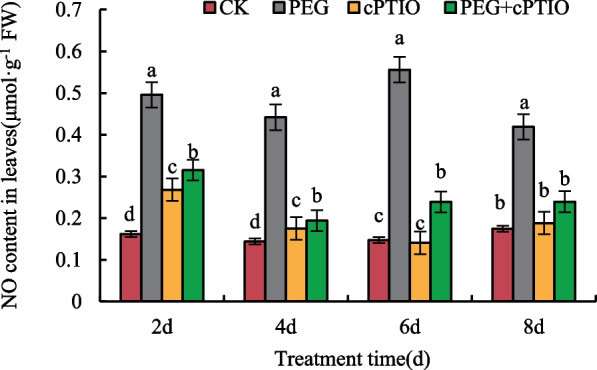
Changes of NO content in leaves of alfalfa seedlings under different treatments. CK: normal water, PEG: Drought stress treatment, cPTIO: Nitric oxide scavenger, PEG + cPTIO: Drought stress and nitric oxide scavenger treatment. Different letters in the same treatment days indicated a significant difference (*P <* 0.05)

NO is an important signaling molecule in plants and plays an important role in stress. To determine the content of NO in alfalfa leaves under different treatments, PEG6000 was used to simulate drought stress and cPTIO was used as a NO scavenger. The result is shown in Fig. 1, the NO content of cPTIO treatment was lower than that of PEG and PEG + cPTIO treatment during the whole treatment period, but higher than that of the control group. The NO content of PEG + cPTIO treatment decreased at the initial stage of stress but increased slightly at the later stage of stress. Compared with PEG treatment, the NO content of PEG + cPTIO treatment was significantly reduced at all time periods.

### Changes of ABA, GA3, IAA, and SA contents in leaves of alfalfa seedlings

**Fig. 2:**
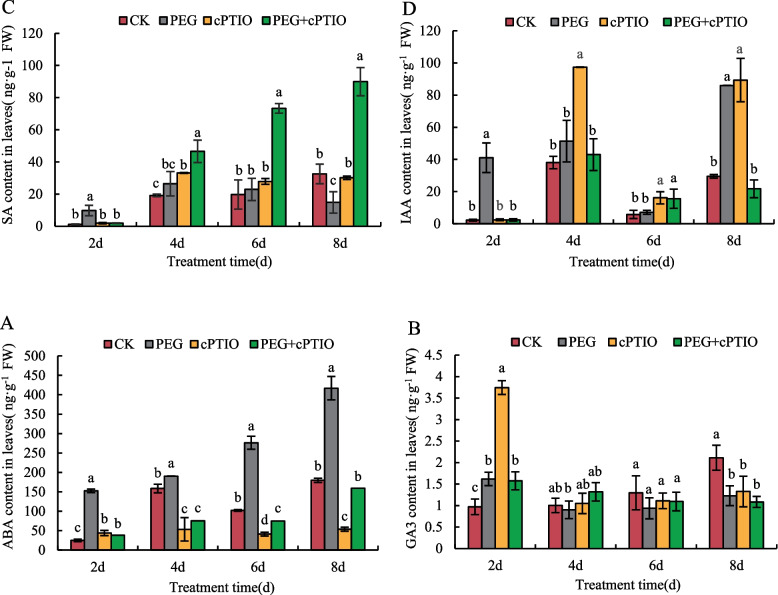
Changes of ABA, GA3, IAA, and SA contents in leaves of alfalfa seedlings. **A** ABA content, **B** GA3 content, **C** SA content, and **D** IAA contents of alfalfa in leaves. Different letters in the same treatment days indicated a significant difference (*P <* 0.05)

To investigate the response of endogenous hormones to NO in alfalfa seedlings under drought stress, the contents of ABA, IAA, SA, and GA3 in alfalfa leaves were determined. As shown in Fig. 2A, ABA content in alfalfa leaves of all treatments reached the maximum value on the 8th day, and ABA content in PEG treatment was higher than that in other treatments at all periods, but the ABA content in PEG + cPTIO treatment was 61.76% lower than that in the PEG treatment. The GA3 content in alfalfa leaves was shown in Fig. 2B. With the extension of drought stress time, except CK, the GA3 content in alfalfa leaves under other treatments showed a decreasing trend with the extension of stress time, and the GA3 content in alfalfa leaves under other treatments reached the maximum value on the second day, among which the GA3 content in cPTIO treatment was the highest. The GA3 content of PEG + cPTIO treatment gradually decreased during the whole stress period, but there was no significant difference between PEG treatment and PEG + cPTIO treatment during the whole treatment period. As shown in Fig. 2C, the SA content in alfalfa leaves treated with PEG initially increased and then decreased with the extension of stress time, but the SA content in PEG + cPTIO treatment was higher than that in other treatments during the whole treatment period (except the second day), and increased with the extension of treatment time. Under drought stress, the IAA content in alfalfa leaves of all treatments showed a trend of first increasing, then decreasing and then increasing, among which IAA content in cPTIO treatment reached the maximum value in 4d (Fig. 2D). Except the 6th day, the IAA content of PEG treatment was higher than that of PEG + cPTIO treatment, and the IAA content of PEG treatment reached the maximum value on the 8th day, while that of other treatments reached the maximum value on the 4th day.

### Deep sequencing results of alfalfa small RNA (sRNA) libraries

To reveal the miRNAs associated with drought and endogenous NO in alfalfa leaves, 12 small RNA libraries were constructed and sequenced. These samples included three untreated alfalfa leaf sample repetitions (CK), three drought-treated alfalfa leaf sample repetitions (PEG), three nitric oxide scavenger treated alfalfa leaf sample repetitions (cPTIO), and three drought and nitric oxide scavenger treated alfalfa leaf sample repetitions (PEG + cPTIO). Through correlation analysis of samples, sRNA libraries of 9 samples were obtained for subsequent biological analysis. The number of original reads and clean reads obtained by sequencing is shown in Table 1. By extracting both the adapter and low amount of reads, the four samples obtained 23.52 million (accounting for 87.63% of raw reads), 18.05 million (accounting for 74.41% of raw reads), 21.32 million (accounting for 88.68% of raw reads) and 18.93 million (accounting for 81.31% of raw reads) clean reads were used for subsequent biological analysis, in which the reads compared to the miRNAs on the reference genome in the 4 (CK, PEG, cPTIO, and PEG + cPTIO) samples reached 15.69%, 13.48%, 15.65%, and 15.45% in the whole original data, among which the number of reads compared to the other two samples was more in CK and cPTIO.

**Fig. 3:**
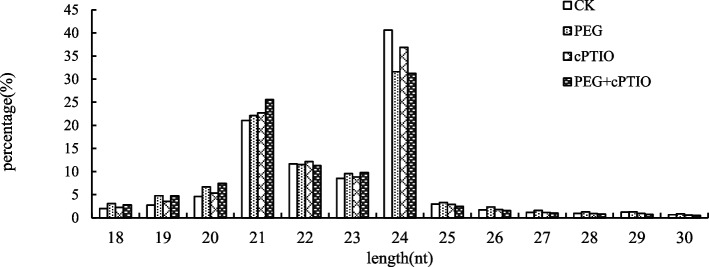
Sequence length distribution of small RNAs in alfalfa four libraries

sRNA of different lengths had different functions. 21/22nt sRNA was mainly related to post-transcriptional gene silencing and mRNA cutting, while 24nt sRNA was mainly related to transcriptional gene silencing and RNA-guided DNA methylation, and most new miRNAs are 24nt in size [16]. As can be seen from Fig. 3, CK, PEG, cPTIO, and PEG + cPTIO samples showed similar variation patterns. miRNAs in the four samples were mostly concentrated in the length of 21-24nt, among which the sequence of 24nt was the most abundant, followed by 21nt and 22nt. PEG + cPTIO was the most abundant in 21nt, while CK and cPTIO were more abundant in 24nt than other treatments.

**Table 1:** Small RNAs profiling and classification in alfalfa

	CK		PEG		cPTIO		PEG + cPTIO	
Category	Count	Percentage	Count	Percentage	Count	Percentage	Count	Percentage
Raw reads	26,849,326	100.00%	24,252,135	100.00%	24,042,562	100.00%	23,283,240	100.00%
Clean reads	23,528,741	87.63%	18,046,004	74.41%	21,321,132	88.68%	18,931,858	81.31%
Mapped to genome	4,212,305	15.69%	3,268,713	13.48%	3,762,635	15.65%	3,597,815	15.45%
rRNA	2,931,163	10.92%	3,527,200	14.54%	3,188,575	13.26%	2,725,769	11.71%
snoRNA	3746	0.01%	7001	0.03%	6017	0.03%	6462	0.03%
tRNA	234,634	0.87%	333,859	1.38%	258,790	1.08%	279,589	1.20%
Repbase	85,207	0.32%	64,750	0.27%	90,427	0.38%	82,073	0.35%
Unannotated	20,273,991	75.51%	14,113,193	58.19%	17,777,321	73.94%	15,837,961	68.02%

### Identification of conserved miRNAs in alfalfa

**Fig. 4:**
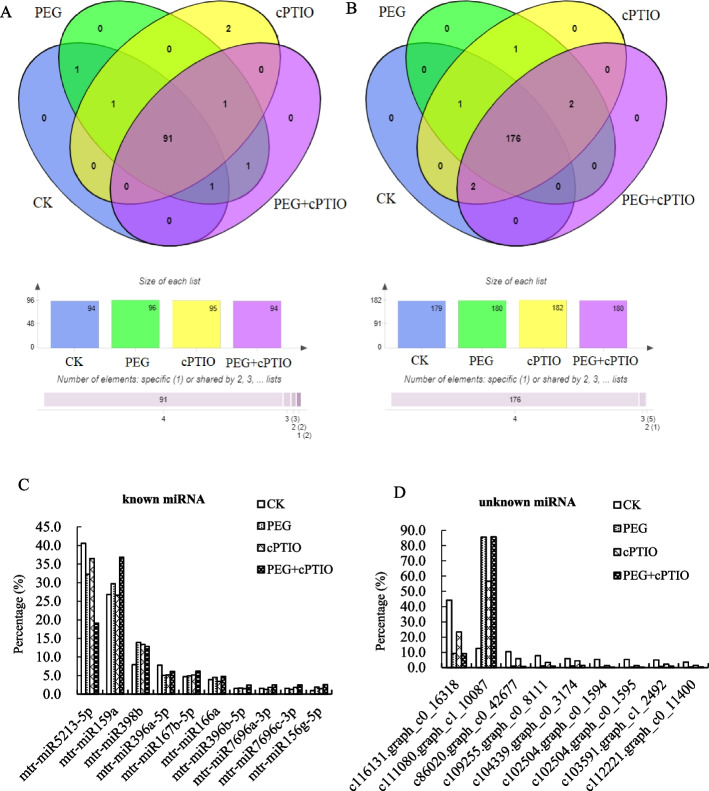
Identification of conserved miRNA and unknown miRNA in alfalfa leaves. **A** miRNAs were known in 4 samples, **B **Identification of unknown miRNAs in 4 samples, **C** Known miRNA with high expression, and **D** Highly expressed miRNAs in unknown miRNAs

By comparing known plant miRNAs in miRBase, we identified some conserved miRNAs obtained by high-throughput sequencing. We found 94, 96, 95, and 94 known miRNAs from CK, PEG, cPTIO, and PEG + cPTIO samples, as shown in Fig. 4A. Among them, 91 miRNAs were identified as conserved miRNAs with certain expressions in 4 samples, and they were from 47 miRNA families respectively. We screened out 10 highly expressed miRNA families from 91 conservative miRNAs, in comparison with other miRNA family readings, miR5213-5p, and miR159a showed the highest levels. The abundance of conserved miRNAs of the top 10 most highly expressed in all libraries is shown in Fig. 4C.

### Identification of new miRNAs in alfalfa

To evaluate unknown miRNAs in clean reads, miRDeep2 was used to predict new miRNAs. A total of 176 new miRNAs were identified by software analysis from 61 miRNA families. 179, 180, 182, and 180 unknown miRNAs were identified in CK, PEG, cPTIO, and PEG + cPTIO samples, respectively, as shown in Fig. 4B. Through analysis, nine new miRNAs with high expression were screened out from 176 miRNAs, as shown in Fig. 4D. Detailed information about the unknown miRNA sequences, the extent of reads, pre-miRNA sequences, genome ID, miRNA length, and Fragments Per Kilobase Million (FPKM) are listed in Table S1. c104339.graph_c0_3174 and c112221.graph_c0_11400 are derived from the miR1507 family, which is unique to legumes and plays a significant role not only in plant growth regulation but also in plant response to adversity.

### The response of miRNAs to drought stress and endogenous nitric oxide under drought stress

To identify miRNAs that respond to drought stress and endogenous NO under drought stress, we compared the expression levels of conserved and novel miRNAs in CK and PEG, CK and PEG + cPTIO, CK and cPTIO, and PEG and PEG + cPTIO samples. The researchers detected 37 miRNAs that responded to drought treatment (including 17 known miRNAs and 20 newly predicted miRNAs, Table S2), the 17 conconservative miRNAs associated with drought were identified as mtr-miR156g-5p, mtr-miR2118, mtr-miR1510a-5p, mtr-miR408-3p, mtr-miR5232, mtr-miR5214-3p, mtr-miR156e, mtr-miR156b-3p, mtr-miR156d-3p, mtr-miR399b, mtr-miR398b, mtr-miR5559-5p, mtr-miR398a-3p, mtr-miR398a-5p, mtr-miR5205a, mtr-miR172a, and mtr-miR2119. In the cPTIO sample, we found 22 differentially expressed miRNAs (including 10 known miRNAs and 12 newly predicted miRNAs, Table S3), of which 13 miRNAs were up-regulated and 9 were down-regulated. A total of 41 miRNAs (including 16 known miRNAs and 25 newly predicted miRNAs, Table S4) were identified in PEG + cPTIO samples that responded to endogenous NO under drought stress. There were 23 up-regulated expressions and 18 down-regulated expressions. In addition, a total of 7 miRNAs (including 2 known miRNAs and 5 newly predicted miRNAs, Table S5), associated with endogenous nitric oxide were identified in PEG and PEG + cPTIO groups, respectively are mtr-miR5213-5p, mtr-miR530, unconservative_c113079.graph_c0_12424, unconservative_c115562.graph_c0_15485, unconservative_c63156.graph_c0_38769, unconservative_c77146.graph_c0_40780, and unconservative_c91116.graph_c0_44142.

**Fig. 5:**
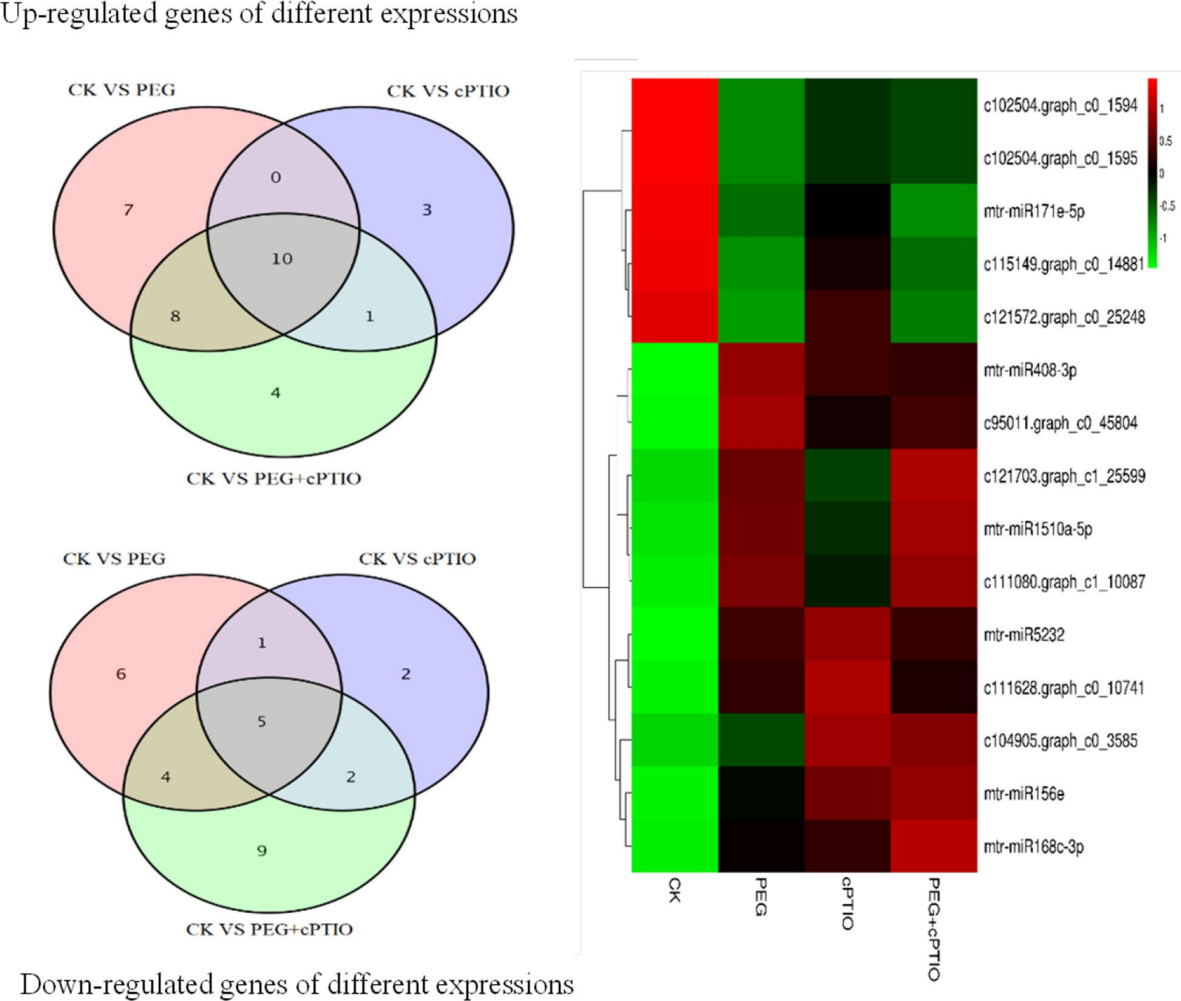
Venn diagram and heat map of differentially expressed miRNAs of 4 samples under drought stress

By analyzing small RNA in 4 samples, we found that 15 miRNAs (10 miRNAs with up-regulated expression and 5 miRNAs with down-regulated expression) were co-expressed in 4 samples of alfalfa leaves (Fig. 5). They come from 11 miRNA families, and their functions and the functions of target genes were shown in Table 2. As can be seen from Fig. 5; Table 2, most miRNAs in alfalfa leaves are up-regulated under drought stress and the addition of cPTIO. Among them, c111080.graph_c1_10087 has a high expression level and its target gene regulates calmodulin, but it is not clear which miRNA family it comes from. In addition, the calcium/calmodulin signaling pathway is involved in a variety of stress responses, such as drought, salt, osmosis, injury, and radiation, all through calmodulin-binding proteins.

**Table 2:** Differentially expressed miRNAs and target gene functions in 4 samples under drought stress

miRNA name	miRNA family	The function of target gene	Reference
mtr-miR1510a-5p	miR1510	NBS class disease resistance genes: Antiviral protein	[23]
mtr-miR156e	miR156	Transcription factors of the SBP family: Plant phase transition and flowering time regulation; Together with plant hormones to control bud development	[24]
mtr-miR168c-3p	miR168	MAPK-miRNA biogenesis and mRNA degradation, Plant development	[25]
mtr-miR171e-5p	miR171_1	GRAS transcription factors - Response to abiotic stress and floral development	[26]
mtr-miR408-3p	miR408	Anthocyanin precursor, cDNA phosphatidylinositol 3 and 4-kinase family proteins, Peptide chain release factor -pollen tube growth	[27]
c121572.graph_c0_25248	miR167_1	Peptide chain release factor-pollen tube growth	[28]
c95011.graph_c0_45804	miR167_2	AUX signaling regulates JA and ABA responses throughout plant development	[29]
c121703.graph_c1_25599	miR396_2	Mycorrhizal and meristem activity; Control cell fate through a variety of plant hormone pathways	[30, 31]

### Prediction and functional analysis of target genes with differentially expressed miRNAs

**Fig. 6:**
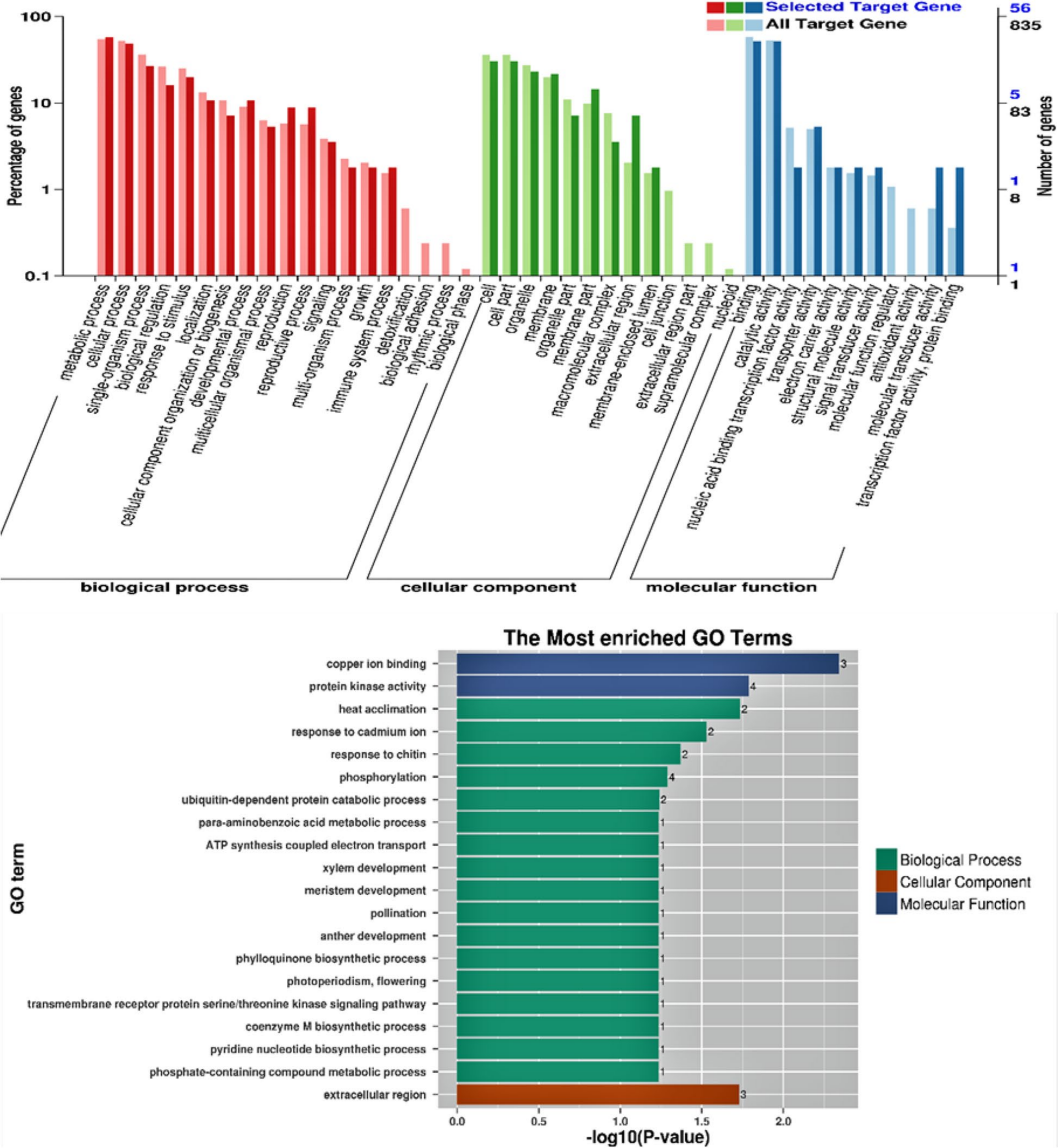
GO functional enrichment analysis of differentially expressed miRNAs in alfalfa

This study used a TargetFinder server to predict the target sequence of differentially expressed miRNAs. To better understand the regulatory role of miRNA in alfalfa, we performed GO analysis on all identified miRNAs and their targets. As can be seen from the GO classification diagram in Fig. 6, targets corresponding to miRNA can be roughly classified into biological processes, cellular components, and molecular functions. Targets mainly focus on biological processes, participating in biological regulation, cellular processes, metabolic processes, responses to stimuli, and single biological processes. For example, thermal adaptation (GO:0010286); Reaction to cadmium ion (GO:0046686); Reaction to chitin (GO:0010200); Phosphorylation (GO:0016310); Ubiquitin-dependent protein catabolism (GO:0006511). The hormone-related biological processes include ethylene biosynthesis (GO:0009693); Stomatal complex morphogenesis (GO:0010103), ethylene-activated signaling pathway (GO:0009873), response to brassinosteroid (GO:0009741), abscisic acid-activated signaling pathway (GO:0009738), defense response (GO:0006952). Most target genes of identified miRNAs target genes that have functions in cells and cell parts, and miRNAs also mainly regulate “binding” genes in molecular functional classification. Such as copper ion binding (GO:0005507) and protein kinase activity (GO:0004672).

**Fig. 7:**
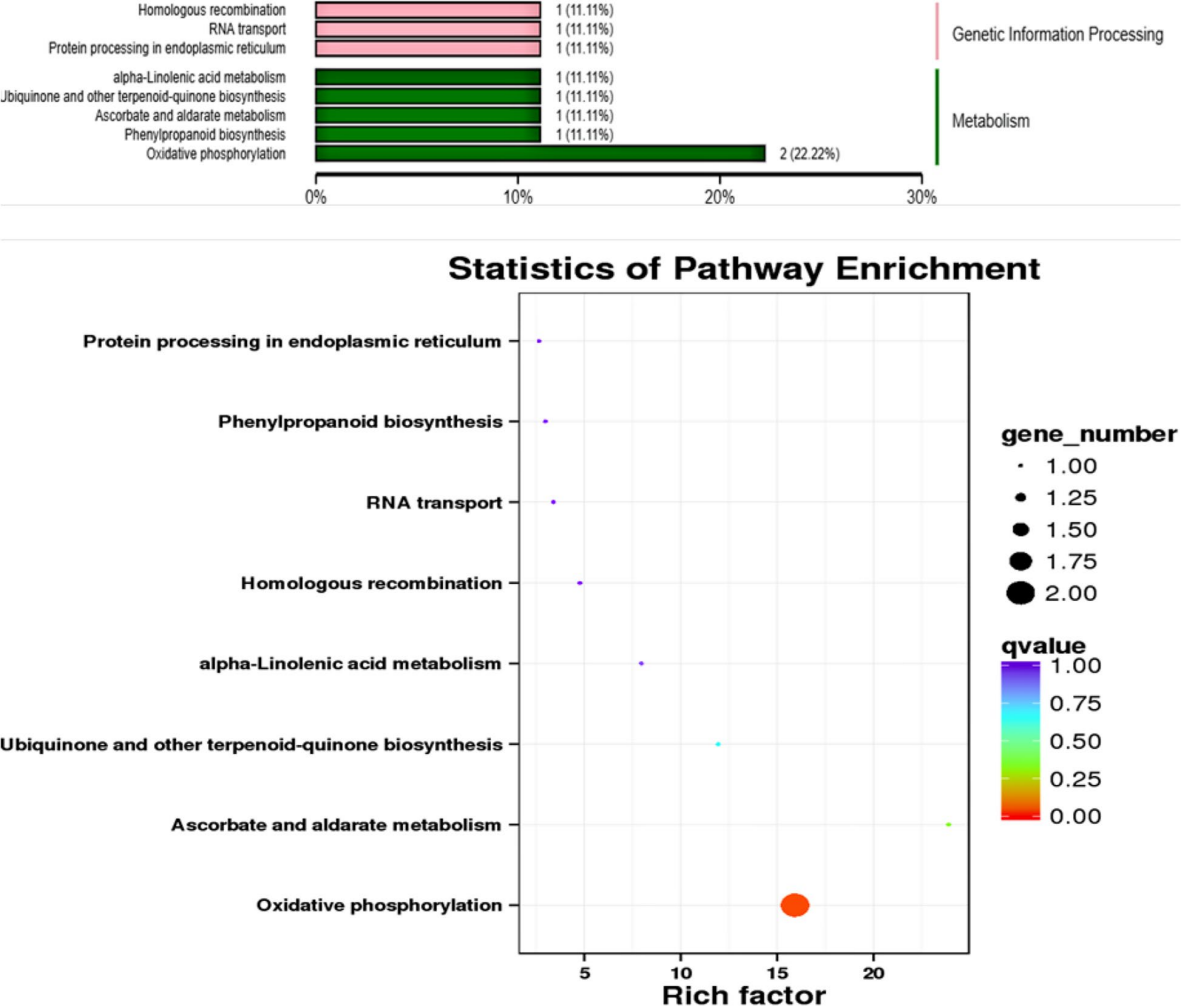
KEGG enrichment analysis of alfalfa pathway differentially expressed miRNAs [37–39]

 As shown in Fig. 7, the differentially expressed miRNAs screened out from the four samples are mainly enriched in 45 miRNAs and 178 targets in KEGG of corresponding target genes, which are mainly enriched in protein processing, homologous recombination, RNA transport, phenylpropane biosynthesis, ascorbic acid, and alginate metabolism, and oxidative phosphorylation in the endoplasmic reticulum. α-linolenic acid metabolism and biosynthesis of ubiquinones and other terpenoids. The most abundant pathway is the oxidative phosphorylation pathway in plants. Among them, miR2118 is involved in phenylpropanoid biosynthesis in alfalfa leaves, c121624.graph_c0_25405 is involved in ascorbic acid and alginate metabolism and L-ascorbic acid oxidase is involved in biosynthesis, transport, and catabolism of secondary metabolites. Under drought stress, c121624.graph_c0_25405 was up-regulated when cPTIO was added. mtr-miR5559-5p and c111080.graph_c1_10087 are involved in the regulation of oxidative phosphorylation, and the target genes regulated by mtr-miR5559-5p are involved in the metabolic process of phosphate-containing compounds and the response to cadmium ions. According to KEGG’s notes, soluble inorganic pyrophosphatase is mainly used to reflect metabolic pathways. The target gene is regulated by c111080.graph_c1_10087 participates in the electron transport of ATP synthesis coupling (GO:0042773). According to KEGG, the subunit of NADH dehydrogenase affects the activity of NADH dehydrogenase (ubiquinone) and quinone bond binding to regulate the metabolic pathway. c115149.graph_c0_14881 is involved in the regulation of α -linolenic acid metabolism. It is involved in the biological process of methylation in plants and affects methyltransferase activity through carboxymethyl transferase of salicylate. c103018.graph_c0_2090 is involved in the biosynthesis of ubiquinones and other terpenoids, mainly in photosynthetic electron transport in photosystem II of chloroplasts (GO:0009772), stomatal complex morphogenesis (GO:0010103) and plastoquinone biosynthesis (GO:0010236) biosynthesis of isoprene diphosphate, methylerythritol 4-phosphate pathway (GO:0019288), regulates its molecular function, 1, 4-dihydroxy-2-naphthalate octandiene ester transferase activity (GO: 0046428).

### Transcriptome and miRNA combined analysis

**Fig. 8:**
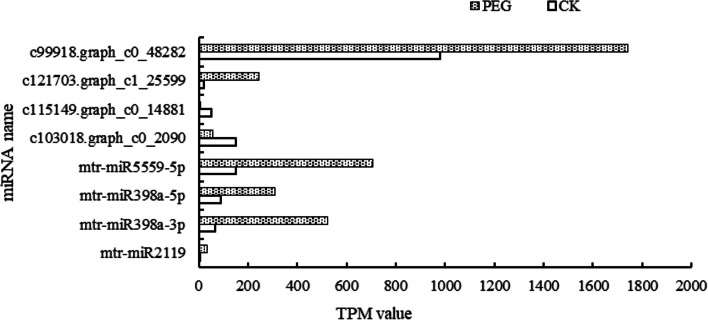
Differentially expressed miRNAs under drought stress in the joint analysis

miRNA negatively regulates the expression of target genes, that is, it inhibits or degrades the expression of target genes after transcription. Through the combination of Small RNA sequencing data and transcriptome data for through-through analysis, not only the utilization rate of sequencing data can be effectively improved, but also the prediction accuracy of miRNA target genes can be improved. By screening differentially expressed miRNAs and target genes (mRNAs), GO enrichment was performed on the predicted target genes, and some hormone-related metabolic pathways were obtained. We also analyzed the KEGG pathway of the target genes with differential miRNA expression, located the target genes mainly enriched in those pathways, and analyzed the target genes with differential miRNA expression and the differentially expressed genes. In log2FC ≥ 1 and *p*-value < 0.05 was used to screen miRNA under drought stress, log2FC ≥ 1 and FDR < 0.01 was the standard to screen mRNA under drought stress. As shown in Fig. 8, most miRNAs are up-regulated under drought stress, among which c103018. graph_c0_2090, mtr-miR5559-5p, and mtr-miR398a-3p have higher expression levels under drought stress. The corresponding target genes of mtr-miR5559 and miR398 families were annotated by GO, KEGG, and NR. It was found that under drought stress, the target genes of mtr-miR398a-3p were involved in the biological process of nucleic acid binding and translation initiation. The target gene of mtr-miR5559 is a disease sieve element protein. The target gene of c103018.graph_c0_2090 is a protein similar to 2-nitropropane dioxygenase, which participates in the regulation of IMP dehydrogenase activity. It can be involved in biological processes including the metabolic process of p-aminobenzoic acid and the response of cadmium ions as well as the biological process of redox process. c103018.graph_c0_2090 and c115149.graph_c0_14881 were down-regulated under drought stress, and their corresponding target genes were up-regulated under drought stress, indicating that miRNA and mRNA were negatively regulated. c103018.graph_c0_2090’s target gene Nr annotation function is F-Box family proteins. The GO function shows that it mainly participates in the following biological processes: respiratory outbreak (GO: 0002679); Reaction to water shortage (GO:0009414); Ethylene biosynthesis process (GO:0009693); Abscisic acid-activated signaling pathway (GO:0009738); Ethylene activated signaling pathway (GO: 0009873); Reaction to chitin (GO:0010200); Thermal adaptation (GO:0010286); Intracellular signal transduction (GO:0035556); These results indicate that the down-regulation of miRNA expression affects the up-regulation of target genes under drought stress, which is involved in plant hormone metabolism pathways. ABA and SA are plant hormones responding to drought stress.

**Fig. 9:**
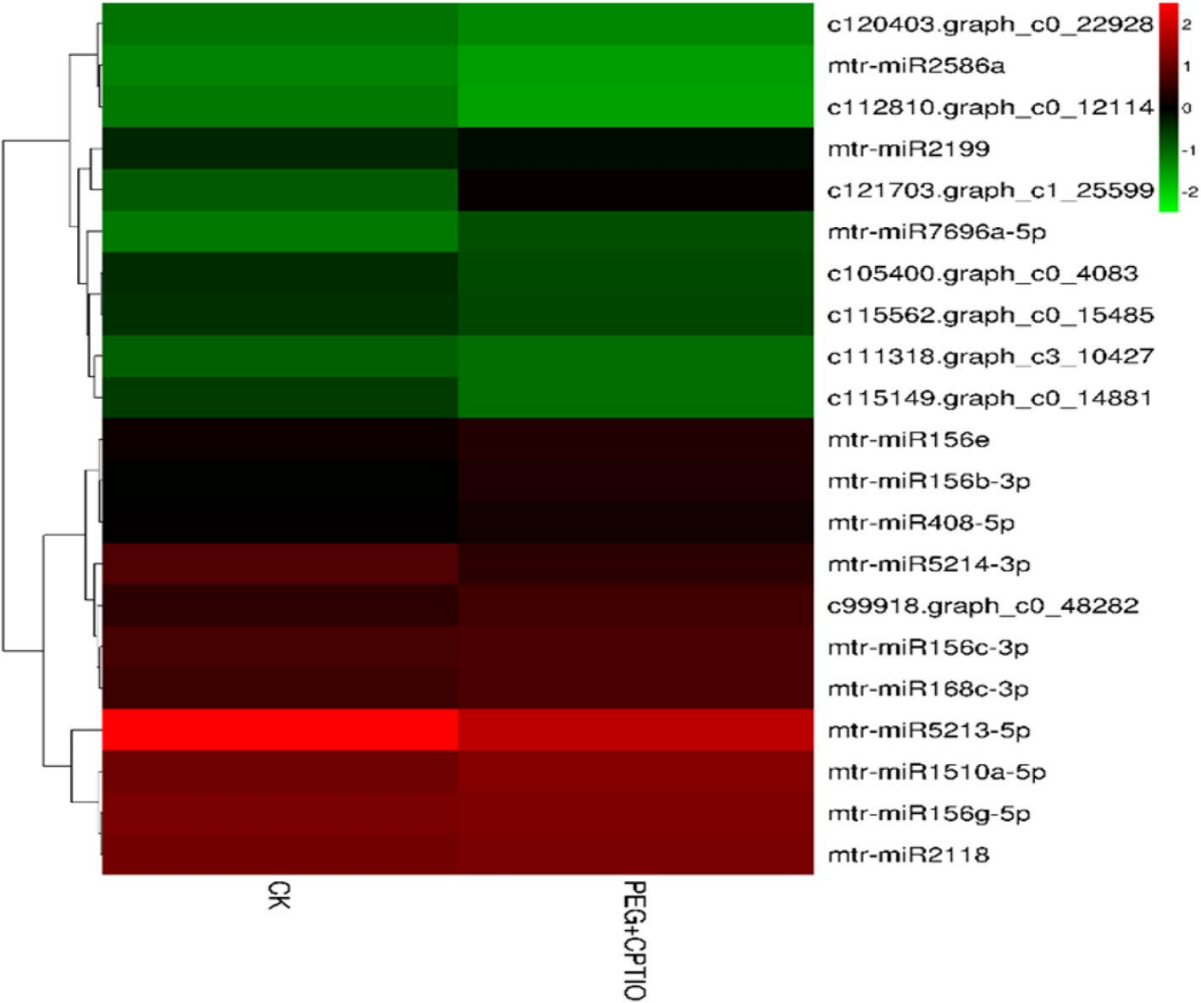
Heat map of differentially expressed miRNA in the joint analysis

 Under drought stress, after the addition of NO scavenger cPTIO, the combined analysis showed that 21 miRNAs were differentially expressed from 16 miRNA families, among which miRNA corresponded to 39 target genes, among which miR156 family played an important role, corresponding to multiple target genes at the same time. Figure 9 shows that most known miRNAs are up-regulated. Among them, miRNA2199 mainly functions as transcription factors, including the BHLH transcription factor and NAI1 transcription factor. Under drought stress, when cPTIO is added, mtr-miR2586a is down-regulated, and its target gene is mainly involved in plant signal transduction. According to GO and KEGG analysis, mtr-miR2586a is mainly involved in the biological process of protein phosphorylation (GO: 0006468), and protein serine/threonine kinase activity (GO: 0004674) to regulate molecular function.

#### Verification of miRNA and its target genes

**Fig. 10:**
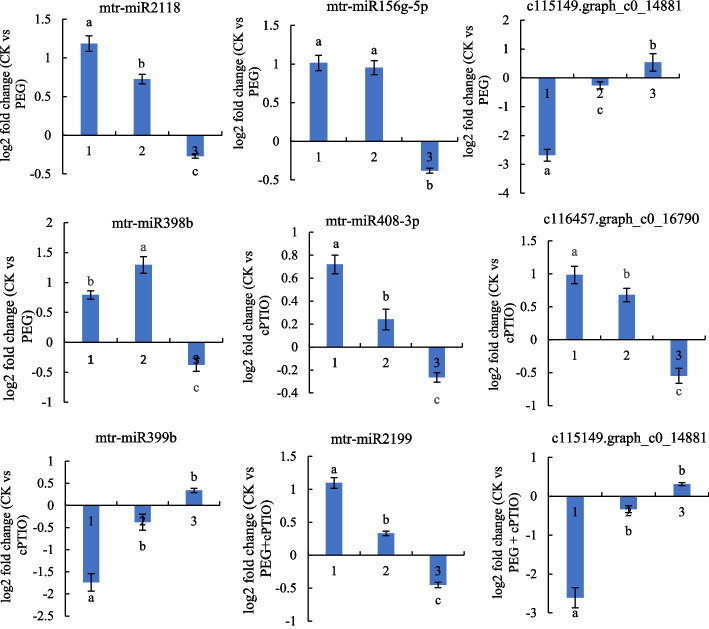
qRT-PCR validation of eight miRNAs and target genes in alfalfa. (1 in the x-coordinate) miRNA expression patterns from small RNA sequencing, (2 in the x-coordinate) miRNA expression patterns from qRT-PCR, (3 in the x-coordinate) target gene expression pattern. Different lowercase letters indicate significant difference (*p <* 0.05)

To verify the expression of miRNAs and their target genes, we selected 8 miRNAs (6 known miRNAs and 2 new miRNAs) and their target genes for qRT-PCR validation. The selected miRNAs are mtr-miR2118, mtr-miR156g-5p, mtr-miR398b, mtr-miR408-3p, mtr-miR399b, mtr-miR2199, not conserved _c115149.graph_c0_14881 and not conserved _c116457.graph_c0_16790. As shown in Fig. 10 and Table S9, the qRT-PCR expression profile was the same as the sequencing results and was contrary to the detected target gene expression pattern. For example, high-throughput sequencing results showed that mtr-miR2188 expression was up-regulated, and the corresponding target gene c10844.graph_c0 expression was down-regulated under drought stress.

## Discussion

In the process of plant growth, drought stress will destroy the cytoplasmic membrane and break the dynamic balance of ions, thus affecting the growth and development of plants [40]. As a bioactive molecule, NO plays an important role in plant growth and development and stress. Studies have shown that NO treatment can promote the growth of wheat roots and above-ground parts under drought conditions, and enhance plant tolerance to drought [41]. In this study, it was found that with the extension of stress time, NO content in alfalfa leaves first increased and then decreased, and reached the maximum on the 6th day. Compared with drought stress alone, cPTIO spray could inhibit the increase of NO content (Fig. 1). ABA is a hormone that changes most obviously in plants under stress and is an important indicator for evaluating the drought resistance of plants [42]. As a signaling molecule, NO is involved in signal transduction in plants and can interact with ABA to participate in plant defense response. In this study, it was found that, except CK, the content of ABA in alfalfa leaves under PEG stress showed an overall trend of increase, and reached the maximum on the 8th day (Fig. 2A). Existing studies have found that exogenous NO treatment significantly promoted the synthesis of endogenous abscisic acid in wheat seedling leaves under salt stress, and NO and ABA signaling molecules had no cumulative effect during the induction process. Meanwhile, the increase of ABA content in leaves could also promote stomatal closure and enhance the drought resistance of plants [43]. In the process of plant root development, IAA synthesized does not stay at a fixed site but is transported to a specific site to play its role [44]. Other plant hormones mostly regulate root development through synergistic or antagonistic effects with IAA [45]. In this study, except on the 6th day of PEG stress, IAA content in alfalfa leaves increased, and reached its maximum value on the 8th day (Fig. 2D). Research observed that drought reduced IAA oxidase activity in wheat leaves, and thus increased IAA content, which was consistent with the conclusion of this study [46]. Studies have shown that exogenous SA can antagonize the effects of drought on fresh root weight and soluble sugar in wheat root, so as to enhance the drought resistance of wheat [47]. This experimental study showed that the SA content in alfalfa leaves treated with PEG + cPTIO gradually increased during drought stress and reached the maximum on the 8th day (Fig. 2C). Klessing [48] et al. found that tobacco leaves treated with NO could rapidly induce an increase in endogenous SA content. In addition, during the process of NO synthesis induced by SA in Arabidopsis Thaliana, the amount of NO synthesis increased with the increase of SA concentration within a certain range and time [49], indicating that the signaling pathways of SA and NO were not isolated, and there was an interaction between them [50]. GA3 is a diterpenoid hormone that promotes cell division and elongation. Studies have shown that drought stress can affect the expression of genes related to gibberellin metabolism in rice roots [51]. The results of this study showed that GA3 content in alfalfa leaves under PEG stress first decreased and then slowly increased (Fig. 2B), and it was found that GA3 may play a negative regulatory signal to slow down growth under plant stress [52], which is consistent with the conclusions of this study.

The current study showed that twelve libraries were constructed from treated with normal watering, drought stress, NO scavenger, and accession NO scavenger under drought stress, and also that three uncorrelated libraries were discarded. All the libraries were sequenced on the Illumina Hiseq 2500 platform. The researchers identified that the known and new miRNAs induced by endogenous NO were under drought stress and drought correlation and their predicted target genes were classified accordingly. According to the sequence, approximately 18–23 million clean reads for each library were generated. Compared to the control, the reads decreased for drought stress samples and other treatments and coatings indicated that the drought treatment and endogenous nitric oxide inhibited the expression of some miRNAs. Thus, more miRNA-related genes and pathways responded to drought stress, which is similar to previous studies on *Medicago sativa* L [53].

The results reported that 94, 96, 95, and 94 known miRNAs from CK, PEG, cPTIO, and PEG + cPTIO groups in alfalfa, respectively. Among them, mtr-miR159a and mtr-miR5213-5p had higher expression levels than other miRNAs. The study also found that mtr-miR398b, mtr-miR396a-5p, mtr-miR167b-5p, mtr-miR166a, mtr-miR396b-5p, mtr-miR7696a-3p, mtr-miR7696c-3p, and mtr-miR156g-5p were the most enriched miRNAs in response to drought stress and endogenous NO. These highly expressed miRNAs may regulate gene expression. It is worth mentioning that mtr-miR5213-5p were the highly expressed miRNAs-this is consistent with Li et al.’s [54] study in alfalfa under natural drought stress. Studies have shown that the miR159 family is interlinked with GA, ABA, and ET in the process of controlling cell death [24]. In addition, alfalfa miR398b was upregulated under drought stress, the target gene was Cu/Zn-superoxide dismutase copper chaperone, a defense against reactive oxygen toxicity. miR396 family has three members, among which, miR396a-5p and miR396a-3p were down-regulated under each treatment, its target gene is growth regulator (GRF), which responds to drought, salt stress, and cell proliferation [55]. At the same time, a total of 176 newly predicted miRNAs from 61 miRNA families were screened out from 4 samples. Among them, miRNAs with high expression mainly come from miR1507 and miR475 families, which also play an important role in plant response to adversity.

Differentially expressed miRNAs were identified by comparing the expression profile of mature miRNAs in PEG and PEG + cPTIO samples with the control group (CK). Different from the expression of miRNAs under natural drought, the results of this study showed that 13 differentially expressed miRNAs were identified in PEG samples, but only 12 were identified under natural drought stress. This slight difference may be caused by the different degrees of osmotic stress simulated by natural drought and PEG. Under drought stress, most miRNAs in alfalfa leaves were up-regulated, and mtr-miR1510a-5p, mtr-miR2118, mtr-miR156g-5p, mtr-miR408-3p, mtr-miR5232 and other miRNAs were up-regulated and highly expressed under drought stress. The miR1510 family mainly targets the genes encoding tobacco Mosaic virus disease resistance proteins, which belong to the NBS class with the most extensive occurrence among the plant disease resistance protein-coding genes [56]. miR2118 is a type of miRNA induced by drought stress, and its corresponding target gene is a nucleotide binding site leucine-rich repeat sequence (NBS-LRR) protein. Jagadeeswaran [57] showed that NBS-LRR domain protein in alfalfa responds to drought, salt, cold, and ABA stress. The expression of miR408 is not only affected by plant growth and development stage but also by environment and abiotic stress. Abiotic stress, such as drought [58], low temperature [59, 60], high salt, ABA stress [61], reactive oxygen species accumulation, and mechanical damage [62], can induce the expression of miR408. Its conservative target gene, the Laccase gene, can also respond to stress. Under drought stress, miR408 increased in alfalfa and barley but decreased in rice and peach [63]. In addition, miR408 is also involved in the regulation of plant secondary metabolism, and overexpression of miR408 can significantly increase anthocyanin content in Arabidopsis thaliana [64]. After the addition of NO scavenger under drought stress, most miRNAs were down-regulated, mtr-miR156g-5p, mtr-miR2118, and c111080.graph_c1_10087 were significantly up-regulated, and the target genes of miR156 family were mainly transcription factors of SBP family. It promotes plant phase transition and flowering time regulation under drought stress [64]. It has also been suggested that miR156 and miR172 are potential pathways by which plant hormones control the timing of bud development. Studies have shown that mtr-miR2118, mtr-miR2199, and miR156 families are up-regulated under drought stress and when exogenously added cPTIO.

Studies have shown that by analyzing the association between mRNA and miRNA, we can better understand the genes and regulation preferentially expressed in different stages of cotton anther development, the importance of the meiosis stage, and the precise regulation of miRNA participation in this process, which will be valuable for clarifying the mechanism of anther development [65]. The integration of miRNA and mRNA transcriptomes [66] revealed that jasmonic acid response genes may be relevant in enhancing the resistance of other plants to pathogens and environmental stresses. mRNA and small RNA transcripts from different cotton development stages [67] revealed that molecular pathways similar to those found in Arabidopsis thaliana may control fruit branching in cotton [68]. In this experiment, transcriptome and miRNA combined analysis results showed that miRNA expression in alfalfa leaves was up-regulated under drought stress and the expression levels of c103018.graph_c0_2090, mtr-miR5559-5p, and mtr-miR398a-3p were higher under drought stress. The target gene annotation function of c103018.Graph_c0_2090 is an F-box protein, and research analysis shows that F-box protein is involved in many signal transduction pathways, such as plant hormone signal transduction, flower organ development, plant self-incompatibility, photomorphogenesis, and biological clock rhythm regulation [69]. Recently, some studies have shown that F-box proteins play an important role in plant defense response and stress. In Arabidopsis thaliana, the F-Box protein encoded by DOR regulates drought tolerance through the abscisic acid (ABA) signaling pathway [70]. Some F-box proteins (such as LKP1 / ZTL, LKP2 / FKL, and FKF1) mediate photomorphogenesis, circadian rhythm, and flowering time [71–73]. TIR1 F-Box protein has been reported to regulate the stability of Aux/IAA protein, which acts as an auxin receptor in Arabidopsis [74]. Studies have shown that in plants under oxidative stress, reactive oxygen species (ROS, O^2−^) directly or indirectly (via calcium) activate transcription factors and then down-regulate miR398 in response to oxidative stress, and its target gene CSD1/CSD2 (copper/zinc superoxide dismutase) increases, thereby increasing the maintenance of oxidative stress in plants, and NO is involved in this process [75].

Under drought stress, endogenous nitric oxide induced mtr-miR530, unconservative_c113079.graph_c0_12424, unconservative_c63156.graph_c0_38769, unconservative_c77146.graph_c0_40780, and unconservative_c91116.graph_c0_44142 were expressed in response to drought stress. In addition, the expression of mtr-miR5213-5p and unconservative_c115562.graph_c0_15485 was silenced in response to drought stress (Table S5). The new miRNAs are helpful to further understand the regulatory mechanism of endogenous nitric oxide under drought stress, and are also a supplement to existing miRNAs. Under drought stress, the exogenous addition of NO scavenger cPTIO revealed 22 differentially expressed miRNAs, among which 13 genes were up-regulated and 9 genes were down-regulated. miR156e and miR156g-5p were all up-regulated. Its target gene is the SBP Domain protein, which is involved in plant phase transformation and regulation of flowering time. Studies have found that miR156 can regulate plant stress tolerance by regulating the Squamosa promoter binding protein (SPL) gene. This gene plays a role in anthocyanin biosynthesis [76]. miR2199 mainly functions in the form of transcription factors, including the BHLH transcription factor and NAI1 transcription factor [77]. In plants, the BHLH transcription factor is mainly involved in the regulation of plant growth and development, such as flower morphogenesis, seed germination, stomatal cell differentiation, guard cell formation, and abiotic stress response [78]. In addition, studies have shown that bHLHs often synergistically control the regulation and induction of the synthesis of a variety of secondary metabolites, including terpenoids, alkaloids, phenylpropanoids, and anthocyanins, with other transcription factor family members, and these secondary metabolites play a crucial role in regulating the plant-environment interaction [79]. GhbHLH1 transcription factors isolated from upland cotton can be induced by ABA, drought, and salt stress [80].

## Conclusions

**Fig. 11:**
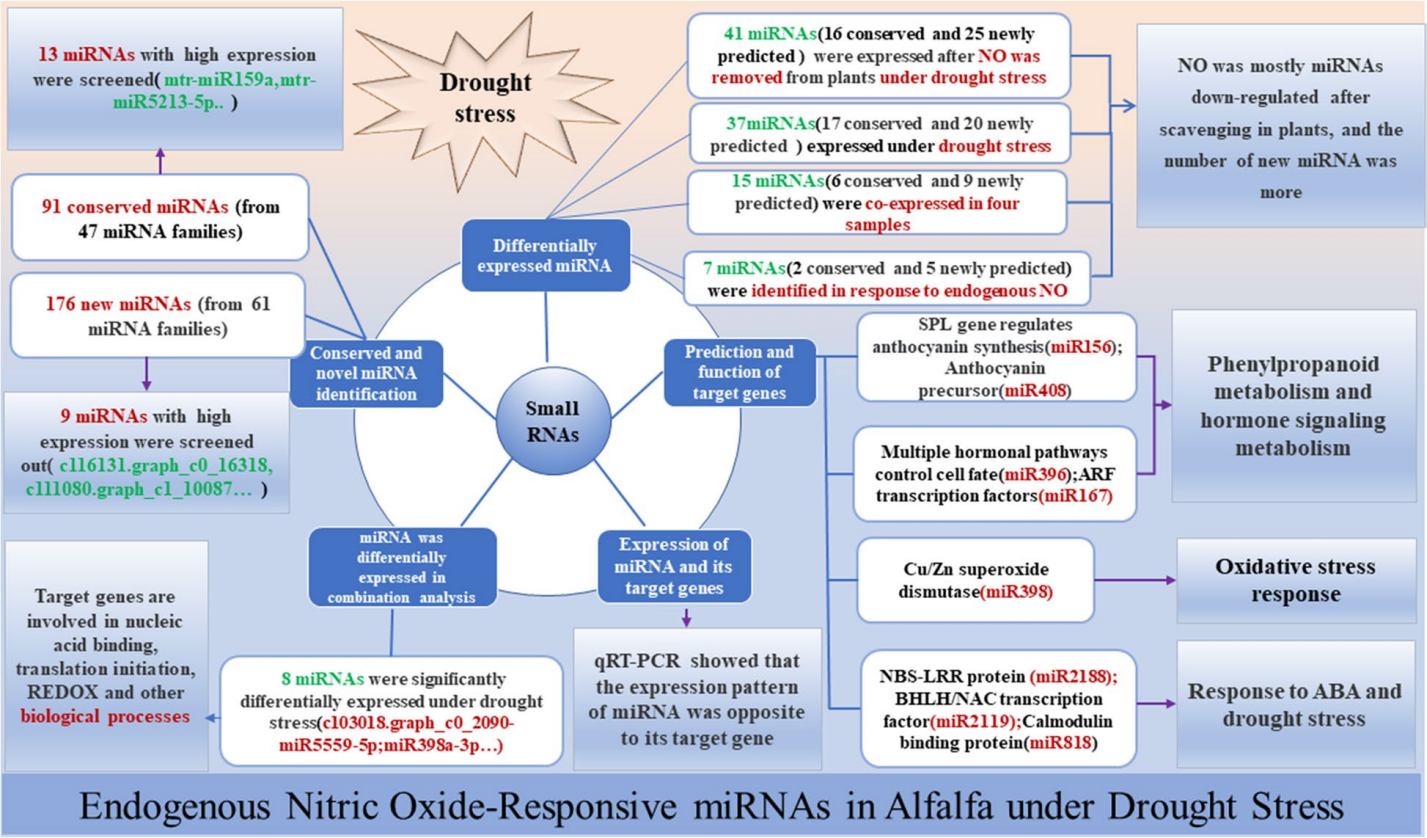
Endogenous Nitric oxide-responsive miRNAs in alfalfa under drought stress

By measuring the contents of four endogenous hormones in alfalfa leaves, it was found that NO could regulate plant growth and stress resistance by inducing the metabolic levels of hormones IAA, ABA, GA3, and SA in alfalfa especially the regulation of ABA and SA in alfalfa. In this study, we found that endogenous NO could regulate plant growth and development through the enrichment of more stress-related biological processes and molecular functions under drought stress. A total of 91 known miRNAs and 176 new miRNAs were identified in four treatments of alfalfa leaves. 37 and 41 differentially expressed miRNAs were identified in PEG and PEG + cPTIO samples, respectively. In addition, 7 differentially expressed miRNAs were identified in PEG and PEG + cPTIO samples, which further supplemented the response of endogenous nitric oxide related miRNAs of alfalfa under drought stress and provided theoretical basis and technical support for molecular breeding and genetic improvement of alfalfa. The four treatments were analyzed and 15 differentially expressed miRNAs with common expression were screened out to predict the function of miRNA target genes. GO enrichment and KEGG pathway analysis showed that endogenous NO in alfalfa leaves could participate in oxidative stress response, hormone, and phenylpropanoid metabolism-related biological processes, and molecular functions in response to drought stress. Transcriptome and miRNA analysis revealed that miR156 and miR2199 were involved in plant stress tolerance in alfalfa leaves (Fig. 11).

## Materials and methods

### Plant materials and growth conditions

*Medicago sativa* L. ‘Sandeli’ was used in this study. Alfalfa plants were grown in pots with a diameter of 15 cm, containing a mixture of nutrient soil and vermiculite (8:2 v/v), in a growth chamber at 25 ± 1℃ under a 12 h light and dark photoperiod. After 30 days of culture, alfalfa seedlings with consistent growth and good development were randomly divided into four groups for treatment. we adopt the Polyethylene glycol-6000 (PEG)was employed to simulate drought stress, along with spraying cPTIO (NO scavenger) externally. The specific test design was CK (normal water), 10% PEG (drought stress), 200 µmol·L-1 cPTIO, and 10% PEG + 200 µmol·L-1 cPTIO. Drought groups were conducted by watering 50 mL of 10% PEG solution once after every 2 days for 7 days. PEG + cPTIO group was sprayed with 5 mL cPTIO solution (tween20 was added into cPTIO treatment solution), once every 24 h under drought stress. The contents of endogenous NO and endogenous hormones (ABA, IAA, SA, and GA_3_) in alfalfa seedling leaves were determined at the 2nd, 4th, 6th, and 8th days after stress, and the determination was repeated 3 times. On the last day, leaf samples of 30 plants from CK, PEG, cPTIO, and PEG + cPTIO groups were collected for high-throughput sequencing. Each treatment group had three biological replicates.

### Determination of endogenous NO content

NO content was determined by the NO kit provided by Suzhou Keming Biological Company (spectrophotometry). The standard curve regression equation was y = 0.016x-0.0103, *R*^2^ = 0.9986.

### Determination of endogenous hormone content

1.0 g of sample material was weighed, rapidly ground with liquid nitrogen, transferred to a 50 mL centrifuge tube, and 10 mL of cooled methanol-formic acid solution (99:1, V/V) was added, placed for 5 min, extracted for 24 h in a 4℃ refrigerator, and centrifuged for 10 min (10,000 r/min) to obtain the supernatant. Absorb 1 mL supernatant, add 4 mL water, mix and shock, and pass through the HC-C18 column (activated with 6 mL methanol and 80% methanol equilibrium column with 5 mL in advance). After loading the column, rinse the C18 column with 10% methanol solution, eluent with the methanol-formic acid solution, collect eluent, and blow nitrogen to nearly dry. The solution of methanol-formic acid was filled to 1 mL, filtered through 0.22 μm, and then determined by liquid chromatic-mass spectrometry (Agilent 1260–6460 LC/MS). The conditions of liquid elution were shown in Table 3. The mass spectrum parameters are shown in Table 4.

**Table 3:** Liquid phase elution conditions of plant hormones

Time (min)	Flow rate (µL·min^−1^)	A solution (%)	B solution (%)
0.00	300	90	10
1.00	300	60	10
2.50	300	55	40
5.00	300	20	45
5.10	300	90	80
7.10	300	90	10

**Table 4:** MS parameters of different endogenous hormones in MRM mode

Plant hormone	Retention time (s)	Mother ion (m/z)	Child ion (m/z)	Fragment or voltage (V)	Collision energy (V)
ABA	2.547	263.1	153.1	90	10
IAA	3.035	174.1	129.9	80	13
SA	3.389	136.9	93.0	70	22
GA_3_	4.010	345.1	239.0	75	9

### Small RNA library construction and sequencing

Trizol reagent (Invitrogen, Carlsbad, CA, USA) was used to separate total RNA from alfalfa leaves treated with CK, PEG, cPTIO, and PEG + cPTIO according to the instructions. RNA integrity was detected by Agilent Bioanalyzer 2100 system (Agilent Technologies, Santa Clara, CA, USA). After ensuring that the sample was tested qualified, 2.5 ng was used as the initial amount of RNA Sample, the volume was supplemented with water to 6µL, and the library was constructed using the Small RNA Sample Pre Kit (NEB, USA). T4 RNA Ligase 1 and T4 RNA Ligase 2 (truncated) were ligated to the 3’ and 5’ ends of the small RNA, respectively. The complementary DNA (cDNA) was synthesized by reverse transcription, and amplified by PCR, and the target fragment was screened by gel separation technique. The 18-30nt fragment was a small RNA library. Qubit 2.0 (Invitrogen, USA) was used to detect the library concentration, and the library concentration was diluted to 1 ng/µL. Agilent 2100 bioanalyzer was used to detect the insert size. The effective concentration of the library was accurately quantified by Q-PCR to ensure the quality of the library. After passing the library the RNA sequences were sequenced on Illumina HiSeq 2500 platform by Biomarker (Beijing, China).

### Identification of conserved and novel miRNAs

Filter out low-quality reads that contain more than 10% of the unknown base, null 50 or 30 adapters, and incorrect sequences shorter than 18nt or longer than 30nt to get Clean Reads. Using Bowtie (version1.0.0) software, clean reads were sequenced with the Silva database (http://gtrnadb.ucsc.edu/), GenBank database (https://www.ncbi.nlm.nih.gov/genbank/), Rfam database (http://rfam.xfam.org/), and Repbase databases (https://www.girinst.org/) eliminate rRNAs, tRNAs, snRNAs, snoRNAs, and other ncRNAs and duplicates. Clean reads were compared with the reference genome (alfalfa transcriptome) and with miRBase and reads whose sequences were identical to those of known miRNAs were considered to be known miRNAs. For unknown miRNA sequences, we can pass miRDeep2 (https://github.com/hangelwen/miR-PREFeR) [81] software to predict new miRNAs in alfalfa leaf. miRDeep2 is a comprehensive software package for the identification of known miRNAs and the prediction of new miRNAs. We used the miRDeep2 software package to align reads aligned to the reference genome with known miRNA precursor sequences in the miRBase database to identify the expression of known miRNAs. At the same time, possible precursor sequences were obtained by alignment of reads to the position information on the genome. Based on the distribution information of reads on precursor sequences (based on miRNA production characteristics, mature, star, loop) and (RNAfold randfold) were evaluated by the Bayesian model to identify new miRNAs. miRDeep2 is mainly used to predict miRNA in animals [81], and miRNA in plants can also be predicted by adjusting parameters and changing the scoring system [82]. The data standardization method, differential expression multiple, and calculation of *p*-value refer to the method of Long et al. [83].

### Differential expression analysis of miRNAs

To identify the differentially expressed miRNAs, DESeq (Version 1.18.0) software was used to analyze the differentially expressed miRNAs between sample groups and obtain the differentially expressed miRNAs set between the four biological conditions. The miRNA counts were normalized as transcripts per million (TPM) with the following formula [83]: TPM = (total number of actual miRNA counts/reads mapped) ×1,000,000, and then log2 ratio and scatter plots were generated by calculating multiple changes and *P*-values based on normalized expressions miRNAs whose expression multiple changes met | log2(multiple changes) | ≥1 and adjusted *p*-value < 0.05 were considered as differentially expressed miRNAs.

### Target gene prediction and functional annotation of miRNAs

Target gene prediction was performed using TargetFinder (Version 1.6) software based on the gene sequence information of known and newly predicted miRNAs and corresponding species [84]. By using BLAST (version 2.2.26) sequence alignment software, the predicted target gene sequence was compared with the database for NCBI non-redundant protein sequences (NR), swiss-port (http://www.uniprot.org/), GO (http://www.geneontology.org/), clusters of orthologous groups (COG) (http://www.ncbi.nlm.nih.gov/COG/), KEGG (http://www.genome.jp/kegg/), protein homologous cluster (KOG) (http://www.ncbi.nlm.nih.gov/KOG/), and homologous protein family (Pfam) (http://pfam.xfam.org/) to obtain the annotation information of the target gene. The detailed information of miRNAs target gene prediction was recorded in Table S8.

### miRNAs and their target genes were analyzed by qRT-PCR

The expression of some miRNAs and target genes was verified by real-time fluorescence quantitative PCR (qRT-PCR). The RNA samples used in the qRT-PCR analysis were the same as those used in high-throughput sequencing. The reverse transcriptase reaction consists of total RNA treated with DNA enzymes and gene-specific stem ring RT primers. Reverse transcription of RNA into cDNA using TUREscript 1st Stand cDNA synthesis (Aidlab biotechnologies Co. Ltd, Beijing, China) kit according to the instructions, miRNAs, and target gene primers are listed in Tables S6 and S7. The following temperature program was used to perform the RT reaction for 3 min at 95℃, 40 cycles of 10 s at 95℃, 30 s at 58℃, and then the holding time was 4 s at 65℃. All reactions were repeated three times. mtr-miR7701-5p and c115758.graph_c0 were used as internal references for miRNA and mRNA, respectively. The relative expression levels of miRNAs and predicted target genes were calculated using the 2^−∆∆CT^ method and the relative expression was displayed as log2FC.

### Supplementary Information

**Additional file 1: Table S1.** Details of miRNA are unknown.

**Additional file 2: Table S2.** miRNAs differentially expressed in CK and PEG samples.

**Additional file 3: Table S3.** miRNAs differentially expressed in CK and cPTIO samples.

**Additional file 4: Table S4.** miRNAs differentially expressed in CK and PEG + cPTIO samples.

**Additional file 5: Table S5.** miRNAs differentially expressed in PEG and PEG+ cPTIO samples.

**Additional file 6: Table S6.** Primers for miRNA datection by RT-qPCR.

**Additional file 7: Table S7.** Primer for target genes detection by RT-qPCR.

**Additional file 8: Table S8.** Target gene information of predicted miRNAs.

**Additional file 9: Table S9.** The expression of miRNAs.

## Data Availability

All data generated or analyzed during this study are included in this published article Additional file. The datasets used and/or analyzed during the current study are available from the authors upon reasonable request ( Qian Ruan, rq1234562022@163.com). The raw data was saved in the National Center for Biotechnology Information (NCBI) sequence read file, and its Sequence Read Archive (SRA) data study deposit number is PRJNA551564.
